# Impairment of Left Ventricular Function in Hyperthyroidism Caused by Graves’ Disease: An Echocardiographic Study

**DOI:** 10.3390/jcm13237348

**Published:** 2024-12-02

**Authors:** Ivana Petrovic Djordjevic, Jelena Petrovic, Marija Radomirovic, Sonja Petrovic, Bojana Biorac, Zvezdana Jemuovic, Milorad Tesic, Danijela Trifunovic Zamaklar, Ivana Nedeljkovic, Biljana Nedeljkovic Beleslin, Dragan Simic, Milos Zarkovic, Bosiljka Vujisic-Tesic

**Affiliations:** 1 Clinic for Cardiology, University Clinical Center of Serbia, 11000 Belgrade, Serbia; jelenapetrovic2212@gmail.com (J.P.); marija3793@gmail.com (M.R.); sonjapetrovic292@gmail.com (S.P.); misa.tesic@gmail.com (M.T.); danijelatrif@gmail.com (D.T.Z.); ivannanedeljkovic@yahoo.com (I.N.); dr.dvsimic@gmail.com (D.S.); 2 Clinic for Digestive Surgery, University Clinical Center of Serbia, 11000 Belgrade, Serbia; bokica978@yahoo.com; 3 Clinic for Endocrinology, Diabetes and Metabolic Diseases, University Clinical Center of Serbia, 11000 Belgrade, Serbia; jzvezdana@yahoo.com (Z.J.); biljana_beleslin@yahoo.com (B.N.B.); milos.zarkovic@gmail.com (M.Z.); 4 Faculty of Medicine, University of Belgrade, 11000 Belgrade, Serbia; bosavt@gmail.com

**Keywords:** hyperthyroidism, Graves’ disease, orbitopathy, left ventricular function, echocardiography

## Abstract

**Background/Objectives:** The thyroid gland has an important influence on the heart. Long-term exposure to high levels of thyroid hormones may lead to cardiac hypertrophy and dysfunction. The aim of the study was to evaluate the morphological and functional changes in the left ventricle in patients with hyperthyroidism caused by Graves’ disease (GD) in comparison with healthy individuals, as well as to investigate potential differences in these parameters in GD patients in relation to the presence of orbitopathy. **Methods:** The prospective study included 39 patients with clinical manifestations and laboratory confirmation of GD and 35 healthy controls. All participants underwent a detailed echocardiographic examination. The groups were compared according to demographic characteristics (age and gender), heart rate and echocardiographic characteristics. **Results:** The patients with hyperthyroidism caused by GD had significantly higher values of left ventricular diameter, left ventricular volume and left ventricular mass compared to the healthy controls. In addition, hyperthyroidism significantly influenced the left ventricular contractility and led to the deterioration of the systolic and diastolic function, as shown together by longitudinal strain, color Doppler and tissue Doppler imaging. However, the patients with GD and orbitopathy showed better left ventricular function than those without orbitopathy. **Conclusions:** Besides the confirmation of previously known findings, our study indicates possible differences in echocardiographic parameters in GD patients in relation to the presence of orbitopathy. Further investigation with larger samples and meta-analyses of data focused on the evaluation of echocardiographic findings in the context of detailed biochemical and molecular analyses is required to confirm our preliminary results and their clinical significance.

## 1. Introduction

Hyperthyroidism is characterized by excessive secretion of hormones by the thyroid gland [1]. The thyroid hormones have systemic effects and lead to a hypermetabolic state with changes in functioning of multiple organs, including the cardiovascular system [2,3,4]. By being essential in energy homeostasis of the heart, these hormones influence not only the frequency and rhythm of the heart, but also myocardial contraction and cardiac output [5].

The thyroid gland produces two main hormones, thyroxine (T4) and triiodothyronine (T3), with the approximate T4:T3 ratio of 4:1, indicating a very high biological potency of T3 [3,4,6]. In addition, the affinity of thyroid receptors for T3 is 10 times greater than that for T4 [2,4,5]. The thyroid axis is regulated by a negative feedback circuit consisting of the hypothalamus, the pituitary gland and the thyroid gland. The hypothalamus secretes thyrotropin-releasing hormone (TRH) which stimulates the pituitary gland to release thyroid-stimulating hormone (TSH) [3,4,5]. TSH activates the thyroid gland to produce and release T4 and T3, which inhibit the secretion of TRH and TSH when being produced in excessive amounts [7]. Another system involved in the functioning and regulation of the thyroid axis is mediated by deiodinases, enzymes which strictly control the intracellular concentrations of thyroid hormones [3,4,5,6,8].

At the cardiac level, the thyroid hormones act through genomic and non-genomic mechanisms [4,7]. By genomic action, T3 controls the transcription of several genes associated with structural and regulatory proteins of the heart [4,5,9,10,11,12]. The long-term exposure to high levels of T3 may increase the synthesis of cardiac proteins, resulting in cardiac hypertrophy and dysfunction. As for the non-genomic effects, T3 mediates changes in various membrane ion channels for sodium, potassium and calcium, as well as in actin polymerization and in a variety of intracellular signaling pathways in the heart and vascular smooth muscle cells [4,5,13,14,15]. All these mechanisms contribute to the maintenance of the normal cardiovascular function, but prolonged exposure to increased levels of thyroid hormones can lead to the opposite effects, causing cardiovascular disorders [4,5,16]. Therefore, it is very important to assess the impact of these hormonal changes on the heart.

Graves’ disease (GD) is the most common cause of hyperthyroidism mediated by autoantibodies against the TSH receptor (TSHR) and the insulin-like growth factor 1 receptor (IGF-1R) [17,18]. The binding of autoantibodies to these target receptors leads to lymphocyte accumulation and the production of proinflammatory cytokines [18]. Therefore, both humoral and cellular immune mechanisms are activated [19]. Besides the thyroid gland, this process affects multiple organs, including the heart [20]. However, thyroid eye disease or Graves’ orbitopathy is the most common extrathyroidal manifestation of GD, occurring in 25% to 50% of patients with GD [18,21,22]. Although usually mild and non-progressive, it has a negative impact on the quality of life and may result in visual loss [22,23]. The complex autoimmune inflammatory process affects orbital fibroblasts, leading to orbital connective tissue swelling due to increased production of glycosaminoglycans, which is followed by orbital remodeling as the consequence of adipogenesis and, if not treated on time, scarring [21].

The presence of TSHRs in the heart has been previously reported [24], indicating shared pathophysiological mechanisms for thyroid and heart disease in GD patients. As for Graves’ orbitopathy, the available data from a pilot study suggest a potential protective role of autoantibodies directed to IGF-1R, since higher levels of these antibodies were detected in GD patients without orbitopathy [25]. On the other hand, it is known that IGF-1 has numerous protective effects on the cardiovascular system [26], which suggests the possibility of additional common pathophysiological mechanisms included in the development of cardiomyopathy and orbitopathy in GD patients.

The literature data have shown that echocardiography is very useful for the detection of structural and functional cardiac changes in patients with hyperthyroidism caused by GD compared to healthy individuals, but still indicate the need for additional research [23]. Previous studies described changes in interventricular septum thickness, end-systolic diameter of the left ventricle and left ventricular mass index, together with changes in ejection fraction, global strain of the left ventricle and left ventricular diastolic dysfunction [27,28,29]. However, data on echocardiographic changes in patients with hyperthyroidism caused by GD regarding the presence of orbitopathy are scarce [30].

The main goal of this study was to conduct an echocardiographic evaluation of the morphological and functional changes in the left ventricle in patients with hyperthyroidism caused by GD in comparison with healthy individuals. Additionally, we aimed to evaluate potential differences in these parameters in patients with hyperthyroidism caused by GD in relation to the presence of orbitopathy to obtain preliminary data in this field.

## 2. Materials and Methods

### 2.1. Study Population

The research was conducted at the University Clinical Center of Serbia in cooperation with the Clinic for Cardiology and the Clinic for Endocrinology, Diabetes and Metabolic Diseases, from January 2018 to December 2022. The prospective study included 39 patients who met the inclusion and exclusion criteria. The inclusion criteria were the presence of clinical symptoms and signs of GD and documented low TSH values, elevated free T3 and free T4 values and the presence of antibodies against TSHR (TSHR-Abs) (Graves group). The exclusion criteria were previously diagnosed cardiac diseases including chronic or acute coronary syndrome, hemodynamically significant valvular diseases, cardiomyopathies, myocarditis, pericardial diseases, congenital heart diseases, as well as pulmonary hypertension, stage 2 or 3 arterial hypertension or a history of uncontrolled arterial hypertension, previous cardiac interventions, pregnancy and any endocrine disorder that could influence the analyzed echocardiographic parameters. The control group consisted of 35 healthy individuals of approximately similar age as those in the examined group, without any documented diseases, with normal values of free T3, free T4, TSH and without TSHR-Abs.

The groups were compared according to demographic characteristics (age and gender), heart rate (HR), and echocardiographic parameters. Additionally, in patients with GD, we analyzed duration of GD, values of TSH and free T4 at the time of examination, presence of orbitopathy confirmed by computed tomography of the orbit, presence of comorbidities and the use of antithyroid drugs and beta-blockers. The study was approved by the Institutional Review Ethical Board (29/II-10, 19 February 2015) and preceded by the acquisition of informed consent from all participants.

### 2.2. Echocardiographic Examination

All patients underwent a color Doppler echocardiographic examination by Phillips ie33 (Phillips, Amsterdam, The Netherlands) or General Electric Vivid 9 (General Electric, Boston, MA, USA) ultrasonic devices with multifrequency probes 2.5–4 MHz. The echocardiographic techniques used for the evaluation were M-mode echocardiography, 2-dimensional (2D) echocardiography, color Doppler echocardiography, tissue Doppler imaging (TDI) and longitudinal strain imaging.

As morphological parameters, we analyzed left ventricular wall thickness (PW), interventricular septum thickness (IVS), left ventricular end-diastolic diameter (EDDLV), left ventricular end-systolic diameter (ESDLV), left ventricular end-diastolic volume (EDVLV), left ventricular end-systolic volume (ESVLV), left ventricular stroke volume (SV) and stroke volume index (SVI), left ventricular cardiac output (CO) and cardiac output index (CI), left ventricular mass (LVmass-ASE), corrected left ventricular mass (LVmass-ASEcorr), left ventricular mass index (LVmass-ASE index), corrected left ventricular mass index (LVmass-ASE corr index), left atrial dimension (LA), left atrial volume index (LAVI) and right ventricle dimension (RV).

Longitudinal strain was used to assess left ventricular contractility in four-chamber view (4A), two-chamber view (2A) and three-chamber view (3A). The global longitudinal strain (GLS) was obtained as the average value of three strain measurements.

As parameters of left ventricular systolic function, we analyzed left ventricular ejection fraction (EFLV), left ventricular fractional shortening (FSLV), lateral and septal mitral annular plane systolic excursion (MAPSE-l and MAPSE-s, respectively), as well as lateral tricuspid annular plane systolic excursion (TAPSE-l). The average value of mitral annular plane systolic excursion (MAPSE-av) was calculated as (MAPSE-l+MAPSE-s)/2. As parameters of left ventricular diastolic function, we analyzed early mitral flow velocity (E), late mitral flow velocity (A), E/A ratio, duration of late diastolic mitral flow (A-wave m-flow), deceleration time of the E-wave of mitral flow (DCT), diastolic filling time of the left ventricle (DFT), duration of a ventricular cardiac cycle (RR interval), DFT/RR interval ratio, velocity time integral of late diastolic mitral flow (VTI A m-flow), velocity time integral of overall mitral flow (VTI m-flow), ratio between the velocity time integrals of late diastolic and overall mitral flow (VTI-A m-flow/VTI m-flow), left ventricular isovolumetric relaxation time (IVRT), left ventricular isovolumetric contraction time (IVCT), left ventricular ejection time (ETLV) and left ventricular myocardial performance index (MPI-LV) calculated as (IVCT+IVRT)/ETLV.

TDI was used to assess the following parameters of left atrial and left ventricular function: early diastolic tissue velocity (e’), late diastolic tissue velocity (a’) and e’/a’ ratio, peak systolic velocity (Vs), ratio between early mitral flow velocity and early diastolic tissue velocity (E/e’), precontraction time (PreCT), contraction time (CT), postcontraction time (PostCT) and myocardial performance index (MPI-TDI) calculated as (PreCT+PostCT)/CT. All these parameters were obtained for the lateral and septal part of the mitral annulus (MA-l and MA-s, respectively), while the average values for the entire mitral annulus (MA-av) were calculated.

### 2.3. Statistical Data Analysis

The statistical analysis was performed using descriptive and analytical statistics. The numerical variables are shown as mean ($\overset{-}{X}$) ± standard deviation (SD) or as median values. The categorical variables are shown as frequencies. The differences between the groups were examined using Student’s *t*-test or the Mann–Whitney test for numerical variables, depending on the normality of distribution as assessed by the coefficient of variation, and the chi-square test for categorical variables. Statistical analyses were performed using the Statistical Software for Social Sciences SPSS 22.0.

## 3. Results

**Figure 1:**
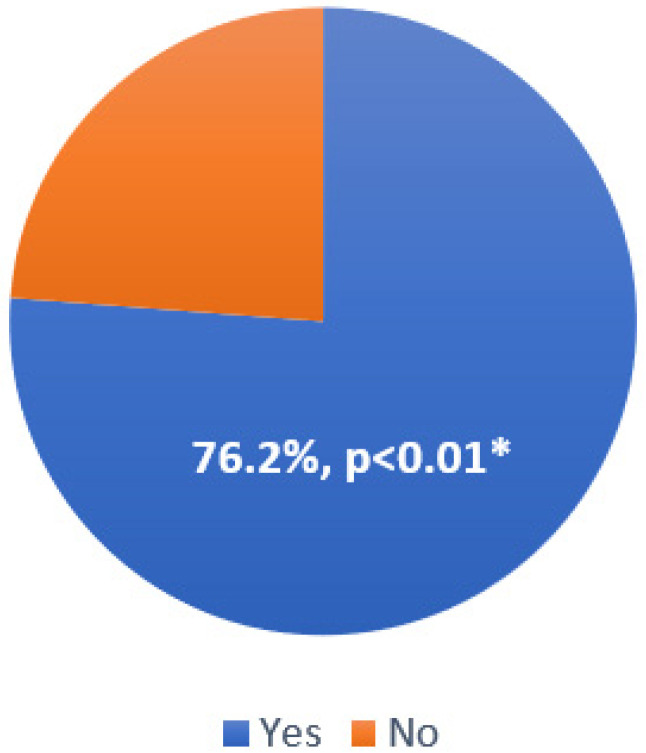
The frequency of orbitopathy in the Graves group; *—Statistically significant difference.

The Graves group included 16 men (41%) and 23 women (59%) with the average age of 53.1 ± 12.6 years, while the control group consisted of 12 men (34%) and 23 women (66%) with the average age of 49.5 ± 12.4 years. The groups did not differ according to either age (*p* > 0.05) or gender (*p* > 0.05). Also, there was no difference in HR between the groups (73 ± 15 vs. 72 ± 12, *p* > 0.05). All patients with GD had it diagnosed previously, with the average GD duration of 4.9 years. The median value of TSH was 0.01 mU/L, and the median value of free T4 was 35.6 pmol/mL. The presence of comorbidities in the Graves group is presented in Table 1. About half of the patients with GD had stage I hypertension, well controlled with medical treatment from the very beginning, and clinically not significant extrasystolic arrhythmia as confirmed by 24 h Holter ECG monitoring. Although more patients from the Graves group had atrial fibrillation, they were still the minority in both groups. The frequency of orbitopathy confirmed with computed tomography of the orbit is shown in Figure 1. All patients with GD continuously used antithyroid drugs and beta-blockers.

**Figure 2:**
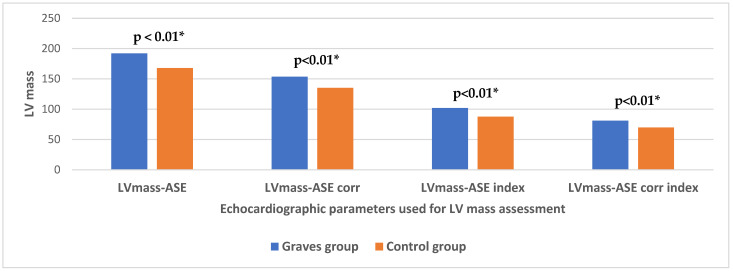
Differences in the left ventricular mass in patients with Graves’ disease and healthy controls; *—Statistically significant difference.

The morphological and functional characteristics of the heart in both groups are presented in Table 2. The patients from the Graves group had significantly higher values of EDDLV, ESDLV, EDVLV and ESVLV compared to the control group. SVI and CI were higher in the group with GD, but without statistical significance compared to the healthy individuals. The left ventricular mass was significantly higher in patients with GD according to all parameters used for its assessment (LVmass ASE, LVmass ASE corr, LVmass ASE index, LVmass ASE corr index) (Figure 2), as well as LAVI, compared to those in healthy individuals. On the contrary, the parameters of the left ventricular systolic function, EF, FS and MAPSE-s were significantly lower in the Graves group compared to the healthy controls.

**Figure 3:**
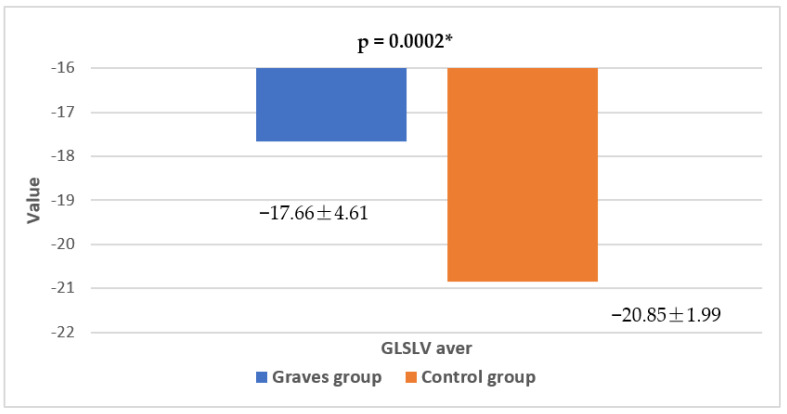
Differences in average left ventricular global longitudinal strain in patients with Graves’ disease and healthy controls; *—Statistically significant difference.

Reduced contractility of the left ventricle in the Graves group was additionally confirmed by GLS, showing significantly lower average values in the Graves group compared to the control group (Figure 3).

The left ventricular diastolic function in the Graves group showed deterioration when compared to that in the healthy individuals, which is presented in Table 3. The Graves group had a significantly lower E/A ratio, indicating slow relaxation of the left ventricle and increased left ventricular filling pressure, which was also supported by a longer duration of A-wave m-flow. On the other hand, these patients also had a longer duration of DCT, DFT, IVRT, IVCT and a shorter duration of ETLV, although it was without statistical significance. The values of MPI-LV were higher to some extent compared to those in the healthy controls, suggesting systolic and diastolic left ventricular function worsening, but without statistical significance.

TDI provided additional quantitative information about the disturbance of the left ventricular diastolic function in the Graves group (Table 4). Higher values of e’-MA-l and e’-MA-av as well as a’-MA-s and a’-MA-l indicated a higher volume load in patients with hyperthyroidism. The obtained value of e’/a’-MA-s < 1 showed left ventricular diastolic dysfunction type 1 in the Graves group. Although e’/a’-MA-l in the Graves group was >1, it was significantly lower in comparison with the value in the control group, which additionally suggested a slower relaxation of the left ventricle among patients with hyperthyroidism. Vs-MA-s and Vs-MA-l were also higher in the Graves group compared to the healthy controls as a consequence of a higher volume load. However, Vs-MA-av was lower as a reflection of left ventricular contractility impairment. The longer duration of PostCT-MA-s, PostCT-MA-l and PostCT-MA-av, as well as the shorter duration of CT-MA-s, CT-MA-l and CT-MA-av in the Graves group compared to the healthy individuals resulted in higher MPI-TDI-MA-s, MPI-TDI-MA-l and MPI-TDI-MA-av, indicating the impairment of both systolic and diastolic left ventricular function in the group with hyperthyroidism.

The comparison of the morphological and functional changes in the heart of the patients with GD depending on the presence of orbitopathy is presented in Table 5. The subgroup of patients with GD and orbitopathy showed significantly higher values of SV (MD: 14.16, 95% CI = [−1.8796–30.1996], *p* = 0.0408), FSLV (MD: 4.09, 95% CI = [−0.4173–8.5973], *p* = 0.0372) and MAPSE-s (MD: 2.05, 95% CI = [−01054–4.2054], *p* = 0.0427), indicating better left ventricular systolic function compared to that in the patients with GD without orbitopathy.

The values of CO, LVmass and EFLV were also higher among the patients with GD and orbitopathy but did not reach the limit of statistical significance.

**Figure 4:**
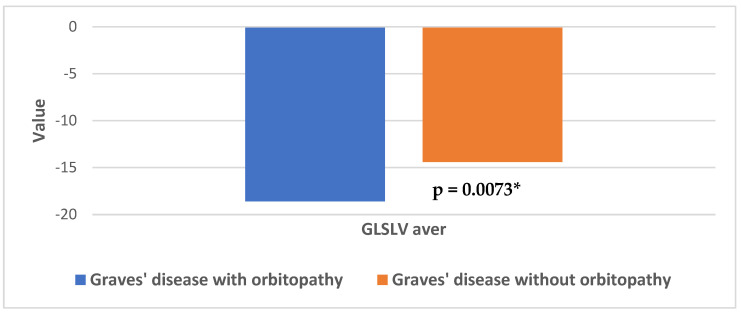
Differences in average left ventricular global longitudinal strain in patients with Graves’ disease in relation to the presence of orbitopathy; *—Statistically significant difference.

The subgroup of patients with GD and orbitopathy also showed higher values of the average left ventricular GLS compared to those without orbitopathy (GLSLV aver: −18.53 ± 3.57 vs. −14.44 ± 6.28, MD:4.19, 95% CI = [0.8763–7. 5037], *p* = 0.0073) (Figure 4).

**Figure 5:**
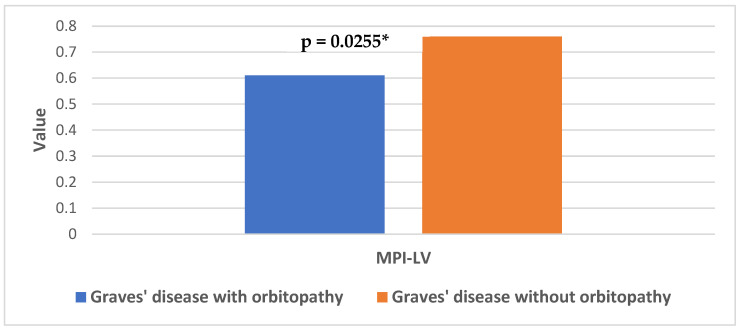
Differences in left ventricular myocardial performance index in patients with Graves’ disease in relation to the presence of orbitopathy; *—Statistically significant difference.

As for the parameters of left ventricular diastolic function, there were also differences between the subgroups of patients with GD depending on the presence of orbitopathy (Table 6). Those with GD and orbitopathy had statistically higher values of VTI m-flow (MD:0.035, 95% CI = [−0.00632–0.06368], *p* = 0.0103), shorter duration of IVCT (MD: 29.18, 95% CI = [8.3856–49.9744], *p* = 0.0193) and lower values of MPI-LV (MD: 0.155, 95% CI = [0.00112–0.30888], *p* = 0.0255), which indicated better systolic and diastolic left ventricular function in this subgroup (Figure 5).

## 4. Discussion

The demographic characteristics in our study did not influence the presented results, since the groups had similar age and gender frequency. However, the literature data indicate that the incidence of left ventricular diastolic dysfunction is higher in patients with overt hyperthyroidism, independently of age, although it increases with aging [31]. The absence of a difference in HR between the Graves and the control group could be explained as the result of beta-blocker treatment. Nevertheless, as previously shown, beta-blocker treatment in patients with hyperthyroidism does not alter the contractile performance [32]. Therefore, HR in our study did not influence the results of the echocardiographic examination. Since hypertension in the Graves group was mild and well controlled from the very beginning, and extrasystolic arrhythmia was not clinically significant, these comorbidities were considered acceptable, without a significant influence on the echocardiographic examination results.

Taking into account that the thyroid hormones, mainly T3, have a direct impact on cardiomyocytes, vascular smooth muscles, the endothelium and the autonomic nervous system, the cardiovascular function is conditioned by the thyroid status [33,34]. Positive inotropic and chronotropic effects of the thyroid hormones on the heart are especially pronounced in hyperthyroidism, where the increase in beta-adrenergic receptors density leads to a greater sensitivity of the cardiac tissue to catecholamines, resembling a state of an increased adrenergic activity. As a consequence, a higher heart rate at rest as well as an increase in blood volume, stroke volume, contractility and cardiac output should be expected [35,36]. Additionally, a decrease in peripheral vascular resistance in hyperthyroidism and the consequent drop in renal perfusion pressure activate the renin–angiotensin–aldosterone system (RAAS), leading to increased renal reabsorption of sodium and water, thus also contributing to increased blood volume, preload and cardiac output [33,37]. Besides the mentioned effects, T3 influences renin and hepatic angiotensin synthesis and contributes to the increased number of angiotensin II receptors in the heart [6]. The described hemodynamic changes cause stretching of the atrial fibers and secretion of atrial natriuretic peptide, leading to further vasodilation and the maintenance of the vicious circle [8]. In addition, T3 increases the synthesis of cardiac proteins, thus causing myocardial hypertrophy in hyperthyroidism [4,8]. The mentioned hemodynamic changes in hyperthyroidism can explain the significant increase in dimensions and volumes of the left heart chambers as well as the increase in left ventricular mass in the patients with hyperthyroidism caused by GD in our study [38]. Considering the previously described genomic effects of T3 on the transcription of structural and regulatory proteins at the myocyte level, which are potentiated during prolonged exposure to high concentrations of thyroid hormones and can lead to pathological hypertrophy and dilation of the heart chambers, the results of our study agree with the literature findings [27]. However, despite the expected increase in contractility in patients with hyperthyroidism, our study showed opposite results compared to those in healthy individuals. Still, our findings are compatible with the previous suggestions that prolonged exposure to excessive amounts of thyroid hormones may lead to contractility reduction [31]. Nevertheless, the positive effects of hyperthyroidism on myocardial contractility and left ventricular systolic function in our study were more pronounced within the Graves group in patients presenting with orbitopathy, as shown by the values of SV, FSLV, MAPSE-s and GLS. Akçay et al. found no differences in left and right ventricular dimensions, ejection fraction and right and left ventricle annular systolic velocities in patients with GD and orbitopathy [30], while no similar literature data are available for comparison of left ventricular contractility assessed by GLS in patients with GD and orbitopathy. Besides a greater volume load and better left ventricular diastolic filling, our findings regarding better contractility and systolic and diastolic function in patients with GD and orbitopathy could be explained by the previously reported common autoimmune pathogenesis of cardiomyopathy and ophthalmopathy in these patients through TSHRs expressed in the human heart [24]. Still, the presented results of our study may be the consequence of different levels of thyroid hormones within the Graves group in relation to the presence of orbitopathy, which were not evaluated due to the small number of patients with GD without orbitopathy. Additionally, IGF-1 has been shown to improve myocardial contractility, stroke volume, cardiac output and ejection fraction [26]. Given the fact that GD patients without orbitopathy have higher levels of antibodies against IGF-1R compared to those with orbitopathy [25] and that our study shows better myocardial function in GD patients with orbitopathy, the question arises of whether the mechanism of action of these antibodies is the same in the orbital and cardiovascular tissue of GD patients, which remains to be elucidated. Moreover, previous animal studies suggested the role of the IGF-1/IGF-1R signaling pathway in cardiac aging and longevity, with a possible delay in senescence-associated myocardial pathologies after deletion of IGF-1R in cardiomyocytes [39]. This also imposes the need for additional studies in the area of IGF-1/IGF-1R signaling among GD patients to evaluate the possible association between cardiac and orbital tissue remodeling, with repercussion on organ function. The understanding of these additional mechanisms involved in the pathogenesis of both orbitopathy and cardiomyopathy in GD patients may have clinical implications in the prediction of heart failure in GD patients in relation to the presence of orbitopathy and, accordingly, in the planning of their treatment.

The hemodynamic changes in hyperthyroidism can lead to a clinical picture of cardiomyopathy with typical symptoms and signs, which may be interpreted as either “de novo” heart failure or the exacerbation of the previously established disease [40]. The association between heart failure and hyperthyroidism varies, as 1–7% of heart failure patients present with hyperthyroidism, and 10–40% of hyperthyroidism patients present with heart failure [41,42]. As a result of the increased heart rate, blood volume, stroke volume, ejection fraction and cardiac output, there is a type of heart failure presentation in hyperthyroidism frequently called "high-output heart failure" [32,43]. Besides the increased activity of RAAS, the excess of thyroid hormones increases the sympathetic system reactivity [44]. Therefore, there is a rise in ventricular filling pressure and pulmonary arterial pressure as well as a rise in sympathetic adrenal activity, which can cause heart failure even in the absence of an underlying cardiovascular disease [7,45,46]. As in the case of other causes of heart failure, hyperthyroidism may manifest as heart failure with reduced EF (systolic heart failure) or with preserved EF (diastolic heart failure).

The majority of patients from the Graves group in our study had heart failure with preserved EF. Since the values of EF were relatively normal among this subgroup of heart failure patients, we suggest using longitudinal strain and speckle-tracking echocardiography as the most sensitive method for the adequate assessment of myocardial contractility whenever possible [47,48,49]. This is particularly important for the assessment of myocardial contractility in the presence of subclinical forms of hyperthyroidism, but also of suspected coronary artery disease, especially considering the possible risk factors associated with the underlying condition [50,51,52]. The results of our study also agree with the literature regarding the left ventricular diastolic dysfunction in hyperthyroidism. Patients from the Graves group had altered duration of PreCT, CT and PostCT, resulting in higher MPI-TDI compared to the healthy controls. All of these parameters support the presence of delayed left ventricular relaxation, increased left ventricular filling pressure and weakened diastolic and systolic function of the left ventricle [4]. TDI in our study appeared to be a very sensitive method for detection of the left ventricular function impairment in patients with Graves’ disease, which is consistent with the findings of Metwalley et al. [29].

The main limitation of this study is the small number of patients. Although it was generally comparable with that of other similar studies conducted so far focusing on a similar topic, the number of patients in our study was particularly small for the subgroup with GD without orbitopathy. Therefore, some of the results are on the edge of statistical significance and require further research with larger samples to be confirmed. Additional studies are also necessary for the investigation of the potential associations between echocardiographic parameters and orbital findings in patients with hyperthyroidism caused by GD, considering more laboratory analyses.

## 5. Conclusions

Hyperthyroidism caused by GD leads to many cardiovascular changes but also affects other organs, including the orbital connective tissue. Echocardiography, in all its modalities, is a noninvasive and widely available diagnostic method for detecting numerous morphological and functional changes in the heart in patients with hyperthyroidism caused by GD. TDI and GLS in our study showed to be the most sensitive echocardiographic methods for the evaluation of patients with GD. Besides being consistent with previously reported data, to the best of our knowledge, this is the first study to report differences in echocardiographic parameters in patients with GD in relation to the presence of orbitopathy. This pilot research draws attention to possible additional shared mechanisms underlying orbital and cardiac changes in patients with GD and orbitopathy, indicating the need for further investigations in this field with larger samples and meta-analyses of data focused on the evaluation of echocardiographic findings in the context of more detailed biochemical and molecular analyses to confirm our results and their clinical significance. Increasing subgroup analyses according to the severity of orbitopathy and thyroid function status as well as performing follow-up studies with these patients would bring more insights regarding the potential significance of our preliminary results.

## Figures and Tables

**Table 1:** The presence of comorbidities in patients with Graves’ disease and healthy controls.

Groups	Graves Group (*n* = 39)	Control Group (*n* = 35)	*p* Value
Variables			
Hypertension, *n* (%)	18 (46.2)	3 (8.6)	**0.00034**
Atrial fibrillation, *n* (%)	3 (7.6)	1 (2.8)	**<0.00001**
Extrasystolic arrhythmia, *n* (%)	21 (53.8)	1 (2.8)	**<0.00001**

Bolded values indicate statistically significant difference.

**Table 2:** Morphological and functional characteristics of the heart in patients with Graves’ disease and healthy controls.

Groups	Graves Group (*n* = 39)		Control Group (*n* = 35)		*p* Value
Variables	$\overset{-}{X}$	SD	$\overset{-}{X}$	SD	
EDDLV (cm)	5.26 ↑	0.51	4.96	0.39	**0.0038**
ESDLV (cm)	3.49 ↑	0.55	3.11	0.37	**0.0048**
PW (cm)	0.79	0.10	0.78	0.13	0.4112
IVS (cm)	0.79	0.10	0.79	0.14	0.4990
EDVLV (mL)	135.14 ↑	32.85	116.93	22.05	**0.0035**
ESVLV (mL)	52.73 ↑	20.29	39.11	11.74	**0.0010**
SV (mL)	83.53	21.42	77.81	15.00	0.0965
SVI (mL/m^2^)	44.12	9.59	41.31	7.17	0.0908
CO (L/min)	6.05	1.85	5.57	1.21	0.0950
CI (L/min/m^2^)	3.27	0.91	2.93	0.59	0.1297
LVmass-ASE (g)	191.93 ↑	48.42	167.91	46.60	**0.0075**
LVmass-ASE corr (g)	153.67 ↑	38.68	135.00	37.42	**0.0091**
LVmass-ASE index (g/m^2^)	102 ↑	21.66	87.66	20.39	**0.0012**
LVmass-ASE corr index (g/m^2^)	81.17 ↑	18.38	69.82	16.48	**0.0010**
EFLV (%)	61.94 ↓	8.96	66.92	5.80	**0.0034**
FSLV (%)	33.73 ↓	6.04	37.2	4.76	**0.0051**
LA (cm)	3.59	0.75	3.35	0.38	0.2449
LAVI (mL/m^2^)	40.50 ↑	27.44	26.73	6.3	**0.0025**
MAPSE-l (mm)	13.92	3.53	14.37	2.43	0.2697
MAPSE-s (mm)	12.04 ↓	2.87	14.17	1.72	**0.0003**
TAPSE-l (mm)	20.42	5.6	19.71	4.30	0.2751
RV (cm)	2.64	0.42	2.60	0.38	0.3482

HR: heart rate; EDDLV: left ventricular end-diastolic diameter; ESDLV: left ventricular end-systolic diameter; PW: left ventricular wall thickness; IVS: interventricular septum thickness; EDVLV: left ventricular end-diastolic volume; ESVLV: left ventricular end-systolic volume; SV: left ventricular stroke volume; SVI: left ventricular stroke volume index; CO: left ventricular cardiac output; CI: left ventricular cardiac output index; LVmass-ASE: left ventricular mass; LVmass-ASE corr: corrected left ventricular mass; LV mass-ASE index: left ventricular mass index; LVmass-ASE corr index: corrected left ventricular mass index; EFLV: left ventricular ejection fraction; FSLV: left ventricular fractional shortening; LA: left atrial dimension; LAVI: left atrial volume index; MAPSE-l: lateral mitral annulus plane systolic excursion; MAPSE-s: septal mitral annulus plane systolic excursion; TAPSE-l: lateral tricuspid annulus plane systolic excursion; RV: right ventricle dimension; the arrows indicate the type of difference in the Graves group compared to the control group; bolded values indicate statistically significant difference.

**Table 3:** Left ventricular diastolic function in patients with Graves’ disease and healthy controls.

Groups	Graves Group (*n* = 39)		Control Group (*n* = 35)		*p* Value
Variables	$\overset{-}{X}$	SD	$\overset{-}{X}$	SD	
E (m/s)	0.7	0.18	0.728	0.13	0.2229
A (m/s)	0.66	0.13	0.61	0.14	0.0760
E/A	1.068 ↓	0.33	1.233	0.34	**0.0196**
DCT (ms)	164.46	50.19	159.84	28.81	0.3211
DFT (ms)	439.87	128.80	426.37	97.82	0.3134
RR interval (ms)	872.12	163.97	834.78	119.48	0.1430
DFT/RR interval	0.554	0.38	0.489	0.073	0.1744
A-wave m-flow (ms)	120.55 ↑	21.31	106.09	26.16	**0.0081**
VTI m-flow (m)	0.154	0.039	0.153	0.028	0.4826
VTI A m-flow (m)	0.057	0.021	0.051	0.013	0.0784
VTI-A m-flow/VTI m-flow	0.351	0.099	0.336	0.078	0.2379
IVRT (ms)	107.28	26.26	96.78	17.9	0.5076
IVCT (ms)	77.51	28.25	70.53	24.65	0.2846
ETLV (ms)	282.39	36.38	289.06	35.52	0.2140
MPI-LV	0.647	0.21	0.58	0.13	0.0704

E: early mitral flow velocity; A: late mitral flow velocity; E/A: ratio between early and late mitral flow velocity; DCT: deceleration time of the E-wave of mitral flow; DFT: diastolic filling time of the left ventricle; RR interval: duration of a ventricular cardiac cycle; DFT/RR interval: ratio between diastolic filling time of mitral flow and duration of a ventricular cardiac cycle; A-wave m-flow: duration of late diastolic mitral flow; VTI m-flow: velocity time integral of overall mitral flow; VTI A m-flow: velocity time integral of late diastolic mitral flow; VTI-A m-flow/VTI m-flow: ratio between the velocity time integrals of late diastolic and overall mitral flow; IVRT: left ventricular isovolumetric relaxation time; IVCT: left ventricular isovolumetric contraction time; ETLV: left ventricular ejection time; MPI-LV: left ventricular myocardial performance index; the arrows indicate the type of difference in the Graves group compared to the control group; bolded values indicate statistically significant difference.

**Table 4:** Mitral annulus—Tissue Doppler imaging in patients with Graves’ disease and healthy controls.

Groups	Graves Group (*n* = 39)		Control Group (*n* = 35)		*p* Value
Variables	$\overset{-}{X}$	SD	$\overset{-}{X}$	SD	
e’-MA-s (m/s)	0.110	0.054	0.114	0.128	0.5028
a’-MA-s (m/s)	0.120 ↑	0.063	0.106	0.128	**0.0214**
e’/a’-MA-s	0.994	0.329	1.162	0.584	0.2187
Vs-MA-s (m/s)	0.136 ↑	0.155	0.109	0.143	**0.0028**
E/e’-MA-s	7.255	3.913	7.464	3.323	0.4065
PreCT-MA-s (ms)	96.297 ↑	48.249	78.656	15.342	**0.0139**
CT-MA-s (ms)	252.378 ↓	38.712	287.406	26.366	**0.0001**
PostCT-MA-s (ms)	115.324 ↑	47.745	76.250	16.510	**0.00001**
MPI-TDI-MA-s	0.842 ↑	0.291	0.539	0.088	**0.00001**
e’-MA-l (m/s)	0.145 ↑	0.069	0.105	0.027	**0.0063**
a’-MA-l (m/s)	0.132 ↑	0.065	0.074	0.021	**0.00001**
e’/a’-MA-l	1.195 ↓	0.502	1.480	0.676	**0.0417**
Vs-MA-l (m/s)	0.128 ↑	0.066	0.108	0.144	**0.00001**
E/e’-MA-l	5.396 ↓	2.660	6.831	2.239	**0.0043**
PreCT-MA-l (ms)	93.882	33.984	83.875	19.758	0.3030
CT-MA-l (ms)	261.632 ↓	30.930	282.718	30.333	**0.0033**
PostCT-MA-l (ms)	96.789 ↑	20.824	77.047	22.65	**0.00001**
MPI-TDI-MA-l	0.731 ↑	0.268	0.582	0.143	**0.0022**
e’-MA-av (m/s)	0.142 ↑	0.082	0.121	0.126	**0.0155**
a’-MA-av (m/s)	0.127 ↓	0.062	0.132	0.166	**0.0065**
e’/a’-MA-av	1.104	0.630	1.321	0.601	0.1285
Vs-MA-av (m/s)	0.110 ↓	0.055	0.133	0.194	**0.0014**
E/e’-MA-av	6.358	3.223	7.040	2.705	0.1052
PreCT-MA-av (ms)	96.597	39.845	81.423	12.201	0.0969
CT-MA-av (ms)	258.222 ↓	31.438	284.662	23.791	**0.0002**
PostCT-MA-av (ms)	105.889 ↑	32.165	77.547	20.317	**0.00001**
MPI-TDI-MA-av	0.780 ↑	0.266	0.558	0.100	**0.00001**

e’: early diastolic tissue velocity; a’: late diastolic tissue velocity; e’/a’: ratio between early and late diastolic tissue velocity; Vs: peak systolic velocity; E/e’: ratio between early mitral flow velocity and early diastolic tissue velocity; PreCT: precontraction time; CT: contraction time; PostCT: postcontraction time; MPI-TDI: myocardial performance index assessed by TDI; MA-s: septal part of the mitral annulus; MA-l: lateral part of the mitral annulus; MA-av: average values for the entire mitral annulus; the arrows indicate the type of difference in the Graves group compared to the control group; bolded values indicate statistically significant difference.

**Table 5:** Morphological and functional characteristics of the heart in patients with Graves’ disease in relation to the presence of orbitopathy.

Groups	Graves’ Disease with Orbitopathy (*n* = 30)		Graves’ Disease without Orbitopathy (*n* = 9)		*p* Value
Variables	$\overset{-}{X}$	SD	$\overset{-}{X}$	SD	
EDDLV (cm)	5.31	0.52	5.07	0.48	0.1172
ESDLV (cm)	3.48	0.55	3.5	0.57	0.4783
PW (cm)	0.79	0.09	0.76	0.11	0.2003
IVS (cm)	0.79	0.11	0.77	0.10	0.3160
EDVLV (mL)	137.97	34.05	125.71	28.16	0.1162
ESVLV (mL)	52.63	20.30	53.08	21.50	0.8965
SV (mL)	86.79 ↑	21.92	72.63	16.27	**0.0408**
SVI (mL/m^2^)	44.92	10.12	41.44	7.42	0.1732
CO (L/min)	6.23	1.93	5.46	1.46	0.1373
CI (L/min/m^2^)	3.32	0.97	3.11	0.71	0.2808
LVmass-ASE (g)	196.59	48.36	176.4	48.01	0.1391
LVmass-ASE corr (g)	157.25	38.67	141.74	38.43	0.1487
LVmass-ASE index (g/m^2^)	103.48	22.34	100.44	20.27	0.3587
LVmass-ASE corr index (g/m^2^)	81.30	19.24	80.69	16.21	0.4660
EFLV (%)	62.93	8.08	58.76	11.38	0.1128
FSLV (%)	34.67 ↑	5.55	30.58	6.84	**0.0372**
LA (cm)	3.59	0.69	3.6	0.99	0.4972
LAVI (mL/m^2^)	39.12	27.42	46.19	28.97	0.4472
MAPSE-l (mm)	14.17	2.85	13.01	5.58	0.9840
MAPSE-s (mm)	12.45 ↑	2.71	10.4	3.10	**0.0427**
TAPSE-l (mm)	20.43	5.60	20.38	5.97	0.4926
RV (cm)	2.63	0.38	2.7	0.58	0.3460

HR: heart rate; EDDLV: left ventricular end-diastolic diameter; ESDLV: left ventricular end-systolic diameter; PW: left ventricular wall thickness; IVS: interventricular septum thickness; EDVLV: left ventricular end-diastolic volume; ESVLV: left ventricular end-systolic volume; SV: left ventricular stroke volume; SVI: left ventricular stroke volume index; CO: left ventricular cardiac output; CI: left ventricular cardiac output index; LVmass-ASE: left ventricular mass; LVmass-ASE corr: corrected left ventricular mass; LV mass-ASE index: left ventricular mass index; LVmass-ASE corr index: corrected left ventricular mass index; EFLV: left ventricular ejection fraction; FSLV: left ventricular fractional shortening; LA: left atrial dimension; LAVI: left atrial volume index; MAPSE-l: lateral mitral annulus plane systolic excursion; MAPSE-s: septal mitral annulus plane systolic excursion; TAPSE-l: lateral tricuspid annulus plane systolic excursion; RV: right ventricle dimension; the arrows indicate the type of difference in the patients with Graves’ disease and orbitopathy compared to those without orbitopathy; bolded values indicate statistically significant difference.

**Table 6:** Left ventricular diastolic function in patients with Graves’ disease in relation to the presence of orbitopathy.

Groups	Graves’ Disease with Orbitopathy (*n* = 30)		Graves’ Disease without Orbitopathy (*n* = 9)		*p* Value
Variables	$\overset{-}{X}$	SD	$\overset{-}{X}$	SD	
E (m/s)	0.69	0.16	0.71	0.23	0.8571
A (m/s)	0.62	0.14	0.55	0.08	0.2087
E/A	1.04	0.29	1.03	0.47	0.7039
DCT (ms)	159.83	33.94	179.88	83.26	0.5028
DFT (ms)	452.40	122.75	398.11	148.56	0.2113
RR interval (ms)	883.00	150.46	835.88	209.11	0.2285
DFT/RR interval	0.58	0.43	0.46	0.11	0.1215
A-wave m-flow (ms)	116.14	22.53	105.87	27.31	0.4760
VTI m-flow (m)	0.16 ↑	0.03	0.13	0.05	**0.0103**
VTI A m-flow (m)	0.05	0.02	0.05	0.02	0.4122
VTI-A m-flow/VTI m-flow	0.34	0.09	0.31	0.13	0.7039
IVRT (ms)	106.83	23.95	108.77	34.54	0.4242
IVCT (ms)	72.43 ↓	19.90	101.61	44.01	**0.0293**
ETLV (ms)	285.68	37.93	271.77	31.98	0.1635
MPI-LV	0.61 ↓	0.25	0.76	0.28	**0.0255**

E: early mitral flow velocity; A: late mitral flow velocity; E/A: ratio between early and late mitral flow velocity; DCT: deceleration time of E-wave of mitral flow; DFT: diastolic filling time of the left ventricle; RR interval: duration of a ventricular cardiac cycle; DFT/RR interval: ratio between diastolic filling time of mitral flow and duration of a ventricular cardiac cycle; A-wave m-flow: duration of late diastolic mitral flow; VTI m-flow: velocity time integral of overall mitral flow; VTI A m-flow: velocity time integral of late diastolic mitral flow; VTI-A m-flow/VTI m-flow: ratio between the velocity time integrals of late diastolic and overall mitral flow; IVRT: left ventricular isovolumetric relaxation time; IVCT: left ventricular isovolumetric contraction time; ETLV: left ventricular ejection time; MPI-LV: left ventricular myocardial performance index; the arrows indicate the type of difference in the patients with Graves’ disease and orbitopathy compared to those without orbitopathy; bolded values indicate statistically significant difference.

## Data Availability

The data are unavailable due to privacy and ethical restrictions.
